# Predicting Appropriate Admission of Bronchiolitis Patients in the Emergency Department: Rationale and Methods

**DOI:** 10.2196/resprot.5155

**Published:** 2016-03-07

**Authors:** Gang Luo, Bryan L Stone, Michael D Johnson, Flory L Nkoy

**Affiliations:** ^1^ School of Medicine Department of Biomedical Informatics University of Utah Salt Lake City, UT United States; ^2^ School of Medicine Department of Pediatrics University of Utah Salt Lake City, UT United States

**Keywords:** Decision support techniques, forecasting, computer simulation, machine learning

## Abstract

**Background:**

In young children, bronchiolitis is the most common illness resulting in hospitalization. For children less than age 2, bronchiolitis incurs an annual total inpatient cost of $1.73 billion. Each year in the United States, 287,000 emergency department (ED) visits occur because of bronchiolitis, with a hospital admission rate of 32%-40%. Due to a lack of evidence and objective criteria for managing bronchiolitis, ED disposition decisions (hospital admission or discharge to home) are often made subjectively, resulting in significant practice variation. Studies reviewing admission need suggest that up to 29% of admissions from the ED are unnecessary. About 6% of ED discharges for bronchiolitis result in ED returns with admission. These inappropriate dispositions waste limited health care resources, increase patient and parental distress, expose patients to iatrogenic risks, and worsen outcomes. Existing clinical guidelines for bronchiolitis offer limited improvement in patient outcomes. Methodological shortcomings include that the guidelines provide no specific thresholds for ED decisions to admit or to discharge, have an insufficient level of detail, and do not account for differences in patient and illness characteristics including co-morbidities. Predictive models are frequently used to complement clinical guidelines, reduce practice variation, and improve clinicians’ decision making. Used in real time, predictive models can present objective criteria supported by historical data for an individualized disease management plan and guide admission decisions. However, existing predictive models for ED patients with bronchiolitis have limitations, including low accuracy and the assumption that the actual ED disposition decision was appropriate. To date, no operational definition of appropriate admission exists. No model has been built based on appropriate admissions, which include both actual admissions that were necessary and actual ED discharges that were unsafe.

**Objective:**

The goal of this study is to develop a predictive model to guide appropriate hospital admission for ED patients with bronchiolitis.

**Methods:**

This study will: (1) develop an operational definition of appropriate hospital admission for ED patients with bronchiolitis, (2) develop and test the accuracy of a new model to predict appropriate hospital admission for an ED patient with bronchiolitis, and (3) conduct simulations to estimate the impact of using the model on bronchiolitis outcomes.

**Results:**

We are currently extracting administrative and clinical data from the enterprise data warehouse of an integrated health care system. Our goal is to finish this study by the end of 2019.

**Conclusions:**

This study will produce a new predictive model that can be operationalized to guide and improve disposition decisions for ED patients with bronchiolitis. Broad use of the model would reduce iatrogenic risk, patient and parental distress, health care use, and costs and improve outcomes for bronchiolitis patients.

## Introduction

Bronchiolitis is inflammation of the bronchioles, the smallest air passages in the lungs, primarily seen in children less than age 2. Within the first year of life, 10% of children are diagnosed with bronchiolitis [1]. By age 2, more than a third of children have had a bronchiolitis diagnosis [2]. Bronchiolitis causes about 71 hospitalizations and 77 emergency department (ED) visits per 1000 infant years [3]. In the United States, each year bronchiolitis incurs around 287,000 ED visits [4], 128,000 hospitalizations [5], and $1.73 billion in total inpatient costs (2009) [5]. For children under age 2, bronchiolitis is the most common cause of hospitalization and represents 16% of all hospitalizations [5-8].

Despite the huge burden of bronchiolitis care, hospitalization decisions are made with insufficient evidence [7,9], resulting in variable admission rates [1,6,9-19]. About 32%-40% of ED patients with bronchiolitis are admitted to the hospital [20-22]. Studies suggest that 20%-29% of these admissions are unnecessary [23,24]. Unnecessary admissions waste health care resources, overwhelm hospital capacity, increase patient and parental distress, introduce iatrogenic risk such as exposure to other infectious diseases, and expose other hospitalized children to the respiratory pathogens of these patients [11,17,25]. As many as 10% of infants affected by bronchiolitis have adverse events while in the hospital [26]. Similarly, about 6% of ED discharges for bronchiolitis are unsafe, resulting in ED return with hospital admission [27] due to inadequate treatment [11]. New approaches are needed to improve ED disposition decision making and reduce unnecessary admissions and unsafe ED discharges.

One method to reduce practice variation and improve clinicians’ decision making for bronchiolitis care is to use clinical guidelines [28-31]. However, existing clinical guidelines for bronchiolitis acknowledge that decisions to admit or to discharge are subjective and rely on variable clinical judgment due to a lack of specific objective thresholds with good evidence [30,31]. Clinical guidelines also do not account for differences in patient and illness characteristics including comorbidities [32] and offer limited improvement in determining ED disposition.

Predictive models are an alternative method to reduce practice variation and improve decision making [20-22,33-35]. Predictive models can present objective criteria supported by historical data for an individualized disease management plan. Using data from previous patient encounters to identify patterns, predictive models [36] can overcome the limitations of clinical guidelines. Predictive models can be incorporated into electronic decision-support tools [37] to support the provisional judgment of clinicians or to trigger clinicians to reconsider their judgment in real time [20]. This is especially useful for physicians who see children infrequently or are junior. Typically when results of predictive models are provided, human experts can make better decisions [38].

**Figure 1:**
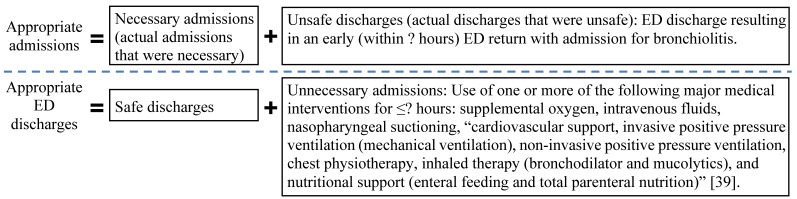
The definition framework of appropriate admission versus appropriate ED discharge that was provided in our previous paper [39]. The details denoted by ""? will be determined by direct evidence in this current study.

As reported in our previous paper [39], existing predictive models for ED patients with bronchiolitis are limited by low accuracy and the assumption that actual ED disposition decisions were appropriate. No operational definition of appropriate admission of ED patients with bronchiolitis exists and no model has been built on appropriate admissions (Figure 1). To fill the gap, we will meet the following 3 aims: (1) develop an operational definition of appropriate hospital admission for bronchiolitis, (2) develop a new model to accurately predict appropriate disposition for ED patients with bronchiolitis, and (3) conduct simulations to estimate the impact of using the model on outcomes.

### Innovations

This study makes the following innovations within the context of bronchiolitis:

1. We will develop a new approach to construct an operational definition of appropriate hospital admission in the ED based on objective data rather than clinical judgment. No such approach currently exists.
2. We will figure out the most important attributes to put into the predictive model using a new simulation method. We will use various attribute combinations to ascertain the minimum requirement on performance and permit tradeoffs for the adaption of our model beyond our setting dependent upon available attributes. Current models are not generalizable beyond the study site because they rely on a certain set of attributes that may be nonexistent in different electronic medical records.
3. We will build the first model to accurately predict appropriate admission for ED patients with bronchiolitis in real time. No such model currently exists. We will transform bronchiolitis care by developing a predictive model to guide appropriate admission for the first time.
4. Our model will increase prediction accuracy by using a rich set of extracted attributes, including known predictors of hospital admission not used in existing models for ED patients with bronchiolitis.
5. Our model will include environmental variables with a potential for a further increase in accuracy. Air quality environmental variables are associated with the daily number of hospitalizations for bronchiolitis [40] and a child’s risk of hospitalization for bronchiolitis within the first year of life [41]. The predictive power of air quality and respiratory virus environmental variables for appropriate admission has never been evaluated.
6. Our study will evaluate the impact of using the model on outcomes. Previous predictive models focused only on accuracy. No impact estimate of using the model on bronchiolitis outcomes has ever been provided.
7. We will use a large data set of 26,701 bronchiolitis patients with high potential to achieve high prediction accuracy. Previous studies are limited by small data sets with typically far fewer than 1000 patients. Many useful predictors of hospital admission cannot be identified in small data sets.

In summary, this study is significant as it will fill gaps by developing a new model to guide and improve disposition decisions for ED patients with bronchiolitis. Broad use of the model will reduce iatrogenic risk, patient and parental distress, health care use and cost, and improve clinical outcomes for bronchiolitis patients. A future study will test the impact of using the model in a randomized controlled trial after implementing the model in an existing electronic medical record to facilitate real-time decision making.

## Methods

Machine learning is a field that studies the automatic improvement of computer algorithms with experience. Machine learning methods—such as support vector machine, neural network, and decision tree—are commonly used in predictive modeling [36] and will be adopted in our study. In comparison to statistical methods, machine learning can improve prediction accuracy, occasionally doubling it, with less stringent assumptions on data distribution [38,42,43].

For all 3 aims, we will use the same patient population and data sets:

1. Patient population: Our study cohort includes children under age 2 who had ED encounters at 22 Intermountain Healthcare facilities for bronchiolitis (ICD-9-CM discharge diagnosis code 466.1 [4]) in the past 10 years. Intermountain Healthcare is the biggest health care system in Utah, comprising 185 clinics and 22 hospitals.
2. Data sets: A large administrative and clinical data set in the enterprise data warehouse (EDW) of Intermountain Healthcare will be used. The Intermountain Healthcare EDW contains a vast set of attributes [44]. Our Intermountain Healthcare data analyst will run SQL queries to obtain a data set that has been de-identified and encrypted and then securely transfer it to a computer that is encrypted and password-protected. Secondary analysis will be conducted on the computer. Intermountain Healthcare has dedicated tables to identify changes in procedure and diagnosis codes. The data set contains electronic documentation of about 85% of pediatric care delivered in Utah [45] and includes approximately 400 attributes. A partial list of categories of these attributes includes: admission date and time; age; orders (eg, medications, labs, exams, immunizations, imaging, counseling, etc), including order name, ordering provider, performing date, and result date; allergies; chief complaint; diagnoses; discharge date; exam result; facility seen for the patient visit; gender; health insurance; health care cost (billed charge, Intermountain Healthcare internal cost and reimbursed cost); height; home address; immunizations; lab test result; language(s) spoken; medication refills; primary care physician as listed in the electronic medical record; problem list; procedure date; procedures; provider involved in the visit; race/ethnicity; referrals; religion; visit type (inpatient, outpatient, urgent care, or emergency department); vital signs; and weight [46].

For the last 5 years, data captured cover more than 2900 patients under age 2 and 3500 ED encounters for bronchiolitis per year. Due to its attribute richness and large size, the data set provides many advantages in the exploration of the proposed predictive models. Furthermore, we will use 21 environmental variables that regional monitoring stations recorded over the past decade within the Intermountain Healthcare region. These variables include carbon monoxide, nitrogen dioxide, particulate matter up to 2.5 μm in size and 10 μm in size, ozone, sulfur dioxide, relative humidity, temperature, precipitation, wind speed, dew point, and activities of each of the following viruses: enterovirus; adenovirus; parainfluenza virus types 1, 2, and 3; human metapneumovirus; influenza A and B viruses; rhinovirus; and respiratory syncytial virus. The data for all nonvirus environmental variables came from federal data sources [47,48], which provide such data throughout the United States. Observation unit admissions will be treated as hospital admissions since the only pediatric observation unit within Intermountain Healthcare has the same admission, coding, billing, and documentation requirements. Our analysis will consider various attribute combinations to ascertain the minimum requirement on performance and will permit tradeoffs for the adaption of our model beyond our setting dependent upon available attributes. Our analysis results will serve as the basis for future expansion of our models to other clinical data sets and diseases beyond bronchiolitis.

### Aim 1: Develop an Operational Definition of Appropriate Hospital Admission for ED Patients with Bronchiolitis

In a recent paper [39], we provided a definition framework of appropriate hospital admissions. As shown in Figure 1, we equate appropriate admissions to necessary admissions and unsafe discharges. We equate appropriate ED discharges to safe discharges and unnecessary admissions. The definition uses several threshold values, such as the maximum number of hours for which major medical interventions are used. Using a data-driven approach, we will fill in these values and develop an operational definition to be used in Aims 2 and 3.

For unsafe discharges, we will examine the distribution of the interval between discharge from the ED and a return visit resulting in admission for bronchiolitis within the period of 2 weeks [49,50]. The 95th percentile of the interval will cover most readmissions and define the return threshold for unsafe discharge. The distribution is highly skewed toward a short interval [27]. Thus, the return threshold will be insensitive to the length of the period chosen.

For unnecessary admissions, we will examine the patients who stayed in the hospital for 12 hours or less and were discharged without readmission for bronchiolitis within 2 weeks. These patients are likely to have been admitted unnecessarily. Their median duration of using major medical interventions (Figure 1) will serve as a conservative threshold for use of major medical interventions in all admissions. Unnecessary admissions are those with major medical intervention exposures for no longer than the threshold. We will conduct sensitivity analysis to evaluate the impact of interactions between major medical interventions and other variables.

If the operational definition for all bronchiolitis patients lacks face validity, we will examine data distributions for different age groups to obtain operational definitions by age group. Since the medical interventions for bronchiolitis have not changed over the last 10 years, we would expect the operational definition to remain the same during this period.

### Aim 2: Develop and Test the Accuracy of a New Model to Predict Appropriate Hospital Admission for an ED Patient with Bronchiolitis

We will use clinical, administrative, and environmental variable attributes to build machine learning models to predict appropriate hospital admission for individual ED patients with bronchiolitis.

#### Data Pre-Processing

Traditional techniques like imputation will be used to handle missing values and identify and correct/remove invalid values [36,51]. In the case of environmental variables, classic methods [40,41] will be used to extract aggregate values (eg, daily average) from raw values. In the case of clinical and administrative attributes, grouper models like the diagnostic cost group system will be used to aggregate diseases, drugs, and procedures to reduce attributes [52].

#### Input Variables

For ED patients with bronchiolitis, predictors of hospital admission have not been exhaustively identified. We compiled in our recent paper [39] a comprehensive list of known predictors. Some of these known predictors (eg, atopic dermatitis [53], low dew point [54], duration of respiratory distress [7], absence of familial atopy [55], enterovirus infection [55], etc) have not been used in existing predictive models for ED patients with bronchiolitis. All known predictors stored in the Intermountain Healthcare EDW and environmental data sets will be used as input variables (ie, independent variables). In addition, our data sets contain attributes beyond known predictors. We will use classic feature selection techniques [56] like the information gain method to find attributes likely to be predictive of appropriate admission. Our team’s clinical experts will review attributes, select attributes with face validity, and add these as input variables. With more new predictors of appropriate hospital admission and larger sample size, we anticipate higher prediction accuracy.

#### Predictive Models

We will use Weka [56] to construct predictive models. Weka is a widely used open-source machine learning toolkit. It integrates a large set of standard machine learning algorithms and feature selection techniques. Both categorical and numerical variables exist in administrative, clinical, and environmental data. Supervised machine learning algorithms that can deal with both categorical and numerical variables, such as *k*-nearest neighbor and random forest, will be used. We will examine each applicable algorithm and tune hyper-parameters manually.

The classic area under the receiver operating characteristic curve (AUROC) [56] performance metric will be used. Our target will be models achieving an AUROC larger than or equal to 0.9, which is considered outstanding discrimination [57]. Some machine learning models, such as decision tree and k-nearest neighbor (ie, similar patients), can be more easily interpreted [58,59]. Other machine learning models, such as random forest, are less straightforward to interpret. If accuracies of models are comparable (AUROC ≥0.9 and ≤0.02 worse for interpretable models compared to less interpretable models), we will favor those that clinicians can more easily interpret.

#### Sample Size Justification and Performance Evaluation

We have 10 years of data. We will train and test predictive models using a standard method. We will perform stratified 10-fold cross validation [56] on the initial 9 years of data to train predictive models and provide estimates of their accuracy. Data from the tenth year will be used to evaluate performance of the best-performing machine learning algorithm, reflecting use in practice. To figure out the environmental variable, administrative, and clinical attributes necessary for high accuracy, we will use backward elimination [36] to remove input variables so long as the AUROC does not decrease by more than 0.02 or go below 0.9.

No AUROC achieved by current care has been reported before. By extrapolating from statistics reported in the literature (unnecessary admissions up to 29% and unsafe ED discharges of 6%), we anticipate the AUROC achieved by current care to be between 0.6 and 0.8+ [20-24,27]. We will test the hypothesis that the model’s prediction will be more accurate by a difference in AUROC of larger than or equal to 0.05. The dependent variable has 2 possible values: appropriate hospital admission and appropriate ED discharge. Assuming a correlation coefficient of 0.6 between the model’s prediction result and the actual disposition decision for both values and using a 1-sided Z-test at a 0.05 significance level, a sample size of 356 instances per possible value of the dependent variable will have 90% power to detect an AUROC increase of 0.05. Data from the tenth year include 3615 ED visits for bronchiolitis, which provides adequate power for testing our hypothesis.

Based on 2 prior studies’ results, we anticipate that our model will achieve an AUROC larger than or equal to 0.9 and outperform current care in making disposition decisions. Neither prior study on predicting a bronchiolitis patient’s ED disposition is similar to our study, which uses appropriate admission as the gold standard. The first study [21] used actual admission as the gold standard and achieved an AUROC of 0.87. The second study [34] used judgment of an attending pediatrician as well as a length of stay longer than 1 day as the gold standard. The predictive model achieved 81% accuracy, better than an average admitting resident’s disposition decision.

For ED patients with bronchiolitis, 17 known predictors of hospital admission (Table 1) are consistently recorded at Intermountain Healthcare facilities and available as structured attributes in our data sets, along with many other potential predictors. We will start building our model using structured attributes. If the model cannot achieve high prediction accuracy, we will extract additional de-identified input variables from ED clinical notes by conducting medical natural language processing on the HIPAA-compliant Homer computer cluster at the University of Utah [60]. For instance, additional input variables include the 8 known predictors of hospital admission (Table 1) that are inconsistently recorded in clinical notes at Intermountain Healthcare facilities.

**Table 1:** The list of known predictors of hospital admission for ED patients with bronchiolitis recorded at Intermountain Healthcare facilities.

Category	Predictors
The known predictors that are consistently recorded at Intermountain Healthcare facilities and available as structured attributes in our data sets	SpO_2_, heart rate, respiratory rate, temperature, age, gender, prior hospitalization, prior intubation, abnormal chest x-ray, low dew point (from the environmental variable data set), rhinovirus infection, coinfection, dehydration, history of bronchopulmonary dysplasia, history of eczema, prematurity, maternal/passive smoking
The known predictor that is rarely recorded as structured attributes at Intermountain Healthcare facilities	enterovirus infection
The known predictors that are inconsistently recorded in clinical notes at Intermountain Healthcare facilities	increased work of breathing, poor feedings, decreased feeding, breastfed, abnormalities on auscultation, retractions, family history of atopy, fewer albuterol in the first hour

If our model still cannot reach high prediction accuracy on the entire group of ED patients with bronchiolitis, we will conduct subanalyses to identify subgroups of ED patients with bronchiolitis on which our model performs well. In this scenario, we will apply our final model only to the identified subgroups of patients. These subgroups are identified by certain characteristics, such as comorbidity, prematurity, age, or ED arrival time (eg, daytime vs night, weekday vs weekend) that are typically independent variables in the original model.

We have large data sets. If scalability is a problem with Weka, a parallel machine learning toolkit like Spark’s MLlib [61] will be adopted to develop predictive models on the secure Homer computer cluster [60].

### Aim 3: Conduct Simulations to Estimate the Impact of Using the Model on Bronchiolitis Outcomes

We will use a method similar to that in Luo et al [46] to establish the model’s utility for future use in clinical practice. More specifically, we will estimate the impact of using the model on bronchiolitis outcomes by applying the model to a retrospective cohort, and determine how the model can be generalized to different sites that collect differing sets of attributes. Our model will be developed using data from Intermountain Healthcare. Our simulations will help determine how to implement the model in other EDs. No prior study has either assessed the impact of using a predictive model on bronchiolitis outcomes or found the set of attributes most essential to generalize the model.

#### Outcomes

We will assess the outcomes of hospital admission, discharge to home, cost, and ED return. The primary outcome is cost. Other outcomes are indirectly reflected in cost and secondary. Each medical claim is companioned by a billed cost, a reimbursed cost, and an Intermountain Healthcare internal cost [52]. The Intermountain Healthcare internal cost [62] will be used because it is subject to less variation resulting from member cost-sharing [52] and more closely reflects actual cost. To deal with inflation, the medical consumer price index [63] will be used to standardize costs to 2014 US dollars. ED returns will be computed using the time interval defining unsafe discharge.

#### Estimate a Model’s Impact

Given a predictive model and a set of input variables, we will estimate the impact of using the model on each outcome. The same method in Aim 2 will be used to train the model on data from the first 9 years. Data from the tenth year have 4 groups: (1) necessary admissions, (2) unnecessary admissions, (3) unsafe discharges, and (4) safe discharges (Figure 1). For each group, we will obtain prediction results, then estimate the outcome if the model’s suggestions were followed. For example, consider necessary admissions. The model will erroneously predict that some of these patients should be discharged. We assume that in clinical application, every such patient will incur an unsafe discharge, an early return visit for bronchiolitis, and a cost equal to unsafe discharges’ average cost. The overall estimated outcome is the aggregate of outcome estimates in all 4 groups. Similarly, we can determine the minimum requirement of the model’s accuracy for the model to be valuable clinically.

#### Sensitivity Analysis

Intermountain Healthcare gathers a vast range of attributes. A different hospital may gather a portion of these attributes. To ensure that the model is generalizable, we will examine miscellaneous attribute combinations and estimate the outcomes of bronchiolitis when using the modified model. Our estimate will determine which attributes are important to include. In the case that an important attribute is nonexistent in a given ED, the estimate can advise substitute attributes that have a minor impact on bronchiolitis outcomes.

Our complete model will include as many as 400 attributes. Conducting simulations for each possible combination of the attributes is not realistic because of the exponential growth of the number of combinations. As an alternative, an attribute grouping approach will be used. This approach associates attributes that commonly coexist based upon the judgment of our clinical experts. If an attribute in a group is not recorded by a hospital, related attributes in the group are also likely to be missing, such as attributes from the same lab test panel. Grouping will allow us to create and publish a table that lists the groups of possible attribute combinations, including bronchiolitis outcomes estimated via simulations and the trained parameters of the predictive model. If a hospital shows interest in implementing the model, the table can help assess expected outcomes in their environment, whether additional attributes need to be gathered, and if so, which ones. One row in the table will reflect the attributes in the PHIS+ [64] data model that standardizes administrative and clinical attributes from 6 major US children’s hospitals. The model of the row will apply directly to at least these 6 hospitals.

#### Sample Size Justification and Performance Evaluation

We will test 3 hypotheses: use of our predictive model will be linked to reduced (1) costs, (2) ED returns, and (3) hospital admissions. Due to their skewed distribution, cost data will be log-transformed [52]. The primary hypothesis will be accepted if the model lowers the log cost by at least 10% of its standard deviation. We will use 1-sided paired-sample t-test to assess the log cost difference between the model’s prediction result and the actual disposition decision. We will use McNemar’s test to assess the difference in ED returns and hospital admissions. A sample size of 857 data instances has 90% power to support the primary hypothesis at a 0.05 significance level. Data from the tenth year include 3615 ED visits for bronchiolitis, which offers sufficient power for testing the primary hypothesis.

If performing simulations on 1 computer is too slow for the numerous combinations of attribute groups, we will conduct parallel simulations on the secure Homer computer cluster [60].

## Results

We have secured institutional review board approvals from Intermountain Healthcare and the University of Utah for this study. At present, we are extracting administrative and clinical data from the Intermountain Healthcare EDW. Our goal is to finish this study by the end of 2019.

## Discussion

The principle of our approach to developing an operational definition of appropriate hospital admission in the ED is general and can be used for other diseases beyond bronchiolitis. Our simulation method will ascertain how a predictive model can be generalized to different sites collecting various sets of attributes, as well as the group of attributes most essential for generalization. This study will use data from a big health care system with numerous heterogeneous facilities spread across a large area. These facilities include EDs at 22 hospitals, ranging from community metropolitan and rural hospitals attended by general practitioners and family doctors with constrained pediatric resources to tertiary care children’s and general hospitals in urban areas attended by subspecialists. Each of these facilities has a differing patient population, scope of services, geographic location, staff composition, and cultural background. This variation creates a realistic situation for identifying factors that are generalizable to other facilities across the United States. One of the models produced during simulation will directly apply to at least 6 large US children’s hospitals. Moreover, this study will produce a new modeling strategy that can be generalized to other clinical conditions where decision making is uncertain.

In summary, our work will transform bronchiolitis care by developing a new predictive model to guide appropriate admission for ED patients with bronchiolitis. Broad use of the model will lower health care use and cost and improve clinical outcomes for bronchiolitis patients. We will have a new simulation method to estimate the impact of using a predictive model on outcomes in dissimilar data environments. The method can be useful for implementing other models.

## Abbreviations

AUROC: area under the receiver operating characteristic curve
ED: emergency department
EDW: enterprise data warehouse
